# Crystal structure of the DNA polymerase III β subunit (β-clamp) from the extremophile *Deinococcus radiodurans*

**DOI:** 10.1186/s12900-015-0032-6

**Published:** 2015-02-27

**Authors:** Laila Niiranen, Kjersti Lian, Kenneth A Johnson, Elin Moe

**Affiliations:** The Norwegian Structural Biology Center (NorStruct), Department of Chemistry, UIT – the Arctic University of Norway, N-9037 Tromsø, Norway; The Macromolecular Crystallography Unit, Instituto de Tecnologia Química e Biológica (ITQB), Universidade Nova de Lisboa, Oeiras, 2780-157 Portugal

**Keywords:** DNA polymerase III β subunit, *Deinococcus radiodurans*, Radiation resistance

## Abstract

**Background:**

*Deinococcus radiodurans* is an extremely radiation and desiccation resistant bacterium which can tolerate radiation doses up to 5,000 Grays without losing viability. We are studying the role of DNA repair and replication proteins for this unusual phenotype by a structural biology approach. The DNA polymerase III β subunit (β-clamp) acts as a sliding clamp on DNA, promoting the binding and processivity of many DNA-acting proteins, and here we report the crystal structure of *D. radiodurans* β-clamp (*Dr*β-clamp) at 2.0 Å resolution.

**Results:**

The sequence verification process revealed that at the time of the study the gene encoding *Dr*β-clamp was wrongly annotated in the genome database, encoding a protein of 393 instead of 362 amino acids. The short protein was successfully expressed, purified and used for crystallisation purposes in complex with Cy5-labeled DNA. The structure, which was obtained from blue crystals, shows a typical ring-shaped bacterial β-clamp formed of two monomers, each with three domains of identical topology, but with no visible DNA in electron density. A visualisation of the electrostatic surface potential reveals a highly negatively charged outer surface while the inner surface and the dimer forming interface have a more even charge distribution.

**Conclusions:**

The structure of *Dr*β-clamp was determined to 2.0 Å resolution and shows an evenly distributed electrostatic surface charge on the DNA interacting side. We hypothesise that this charge distribution may facilitate efficient movement on encircled DNA and help ensure efficient DNA metabolism in *D. radiodurans* upon exposure to high doses of ionizing irradiation or desiccation.

## Background

The bacterial DNA polymerase III β subunit (β-clamp), and the corresponding eukaryotic and archaeal proliferating cell nuclear antigen (PCNA) are ring-shaped proteins that encircle double-stranded DNA. They act as a processivity factor, or a sliding clamp, for a wide variety of proteins that act on DNA including DNA polymerases, DNA ligase, endonucleases and glycosylases (reviewed in [1]). For *Escherichia coli* DNA polymerase catalytic core the replication speed is increased from approximately 20 nt/s with frequent dissociation [2] to approximately 750 nt/s with a processivity of >50 kb in the presence of the β-clamp [3]. To be loaded onto DNA, the sliding clamps need the help of ATP-dependent clamp loaders. Clamp loaders are multi-subunit complexes where ATP hydrolysis is coupled to conformational changes that enable the clamp loader to open the sliding clamp and place it on DNA [4]. Once loaded, the sliding clamp allows the binding of other polymerase subunits.

The crystal structure of a β-clamp was first determined for *E. coli* in 1992 [5], and after that for five other bacteria so far [6-8]. The structures show that the bacterial sliding clamp is a head-to-tail dimer [5], where one of the interfaces is opened by the clamp loader to allow DNA to enter the ring interior [9]. In eukaryotes, the PCNA clamp is also ring shaped but consist of a homotrimer [10] and in archaea such as *Sulfolobus solfataricus* a heterodimer [11]. There are also available two structures of a sliding clamp bound to DNA (*E. coli*, PDB code 3BEP [12] and *S. cerevisiae,* PDB code 3K4X [13]). In spite of the different quaternary structures and a low sequence identity of the different clamp types [14], their overall shape and internal architecture with six similarly folded domains are strikingly similar also compared to bacteriophage clamps. Due to its central role in many DNA related cellular functions the β-clamp is an active target for inhibitor drug design in the development of new antibiotics to combat drug resistant strains [15-17].

In this paper, we describe the crystallographic structure of the DNA polymerase III β-clamp from the extremely radiation resistant bacterium *Deinococcus radiodurans* (*Dr*β-clamp). *D. radiodurans* exhibits an outstanding resistance to ionising radiation and desiccation and tolerates radiation doses up to 5,000 Gray (Gy) without loss of viability whereas most other organisms cannot survive doses above 50 Gy [18]. Such a massive radiation dose is estimated to induce several hundred double-strand breaks (DSB), thousands of single-strand gaps and about one thousand sites of DNA base damage per chromosome (reviewed by [19]). The overall structure of *Dr*β-clamp is similar to *E.coli* β-clamp (*Ec*β-clamp) and consists of a dimer which forms a ring lined by 12 α-helices, with an opening big enough to accommodate dsDNA. Each monomer consists of three domains (A, B, and C) with identical topology. *Dr*β-clamp displays a strong negative electrostatic surface potential on the outside of the ring, but a more even charge distribution inside the ring on the DNA binding surface and in the dimer forming interface. We hypothesise that the evenly distributed surface charge inside the ring helps ensure efficient clamp loading and DNA processivity which are needed to tackle the substantial amount of DNA metabolic processes that are activated upon exposure to high doses of irradiation and desiccation.

## Results and discussion

### Sequence analysis

During the initial part of this work we discovered that the *D. radiodurans* gene sequence *DR_0001* deposited in the GeneBank (Q9RYE8) was incorrectly annotated, encoding a protein of 393 instead of 362 amino acids. This was confirmed by sequence analysis and expression tests. The mistake was most likely caused by the automated gene recognition program used in the annotation of the sequenced genome. These programs can fail to recognise frame shifts caused by insertions or deletions (as demonstrated by [20]). Our discovery is in line with the findings of Baudet et al. [21] who showed that the original annotation of over a hundred *D. radiodurans* R1 genes is wrong and needs to be corrected. In 2014 the *D. radiodurans* R1 genome was re-annotated by the NCBI Ref Seq project, and the new version of *DR_0001* gene product (accession number WP_027480259.1 (GI:653293780), published June 12^th^ 2014) corresponds to our short version of the protein (except for the first Val). The reannotation confirms that we have been working with the biologically relevant version of the protein.

The short *Dr*β-clamp protein sequence shares over 70% identity with other *Deinococcus* β-clamp sequences, and 40 – 70% identity to sequences from other members of the phylum *Deinococcus-Thermus*. Interestingly, the sequence identity to other Gram-negative species is as low as to Gram-positive species, below 32%.

### Overall structure

**Figure 1:**
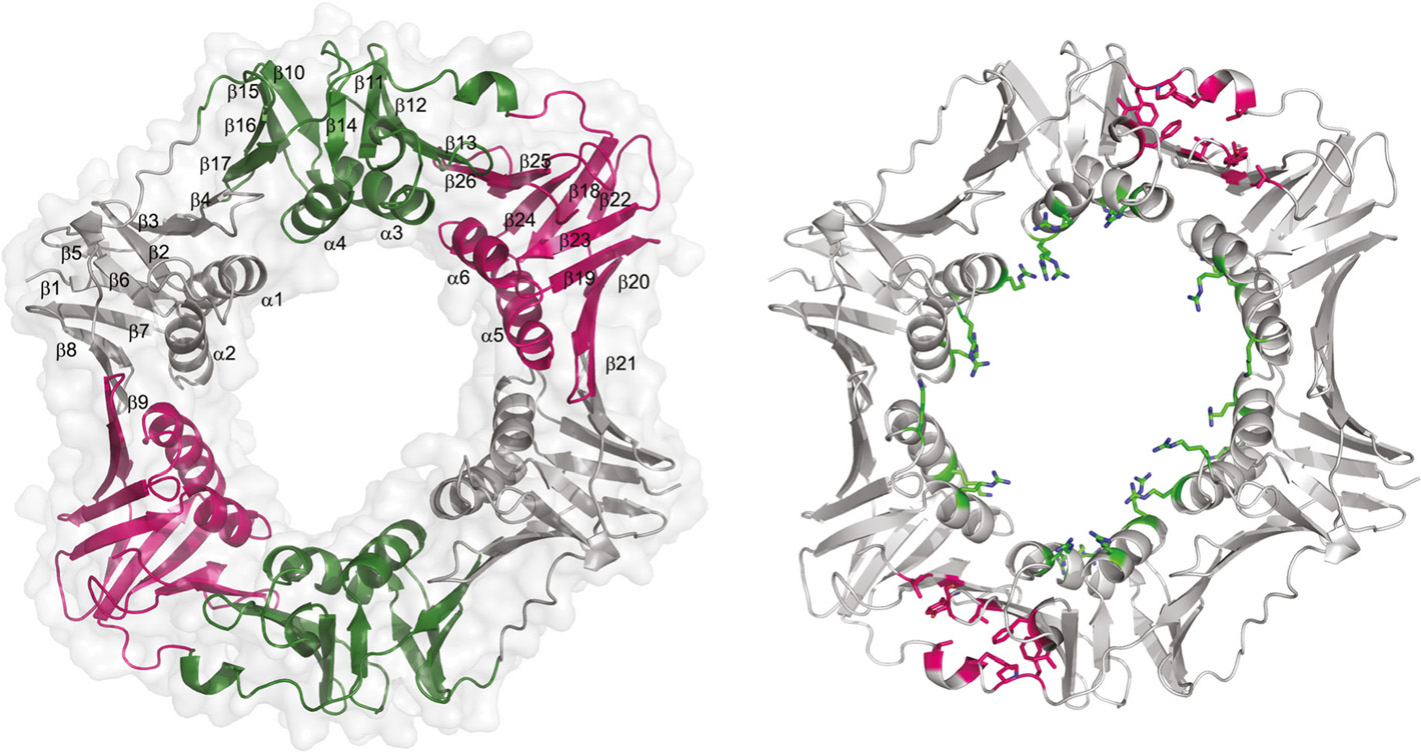
**A schematic model of the** ***Dr*** **β-clamp dimer crystal structure.** In the left panel the secondary structure elements are labelled in chain A, and the molecular surface is shown. Each monomer has three domains (A, B and C) with identical topology, here coloured grey, green and pink, respectively. The right panel shows stick models of the positively charged residues on the ring interior (green), and the residues of the hydrophobic pocket located between domains B and C (pink).

The crystal structure of *Dr*β-clamp was determined in space group P3_2_21 to 2.0 Å resolution using molecular replacement. Despite our efforts, the DNA oligomer the *Dr*β-clamp was co-crystallised with was not clearly visible in the electron density, and could not be modelled into the structure. The asymmetric unit contains one *Dr*β-clamp dimer (residues 1 to 361 of chains A and B), with 292 solvent atoms. A schematic representation of the structural model is presented in Figure 1. The majority (97.7%) of the main-chain torsion angles were in the favoured regions of the Ramachandran plot, with the remaining 2.3% in the allowed regions. The final model had R_work_ and R_free_ values of 19.8% and 23.5%, respectively. The details of the data collection and refinement statistics are given in Table 1.

**Table 1:** **X-ray data collection and crystallographic refinement statistics for** ***Dr*** **β-clamp**

**Data collection**	
X-ray source	ESRF ID29
Space group	P3_2_21
Unit cell (Å)	a = b = 84.41, c = 198.74
Resolution (Å)	30 – 2.00 (2.05 – 2.00)
Wavelength (Å)	0.9724
No. unique reflections	55 287
Multiplicity	3.3 (3.3)
Completeness (%)	98.0 (98.2)
Mean (<I> /<σ_I_>)	17.4 (2.6)
R-sym (%)^a^	4.5 (52.8)
Wilson B-factor (Å^2^)	40.2
**Refinement**	
PDB entry	4TRT
R-factor (all reflections) (%)	20.0
R-free (%)^b^	23.5
Number of atoms	5758
Number of water molecules	292
RMSD bond lengths (Å)	0.008
RMSD bond angles (°)	1.255
Average B-factor (Å^2^)	
All atoms	46.4
Protein	46.2
Water	49.2
Ramachandran plot	
Favoured regions (%)	713 (97.7)
Allowed regions (%)	17 (2.3)
Outliers (%)	0 (0)

Values in parenthesis are for the highest resolution shell.

^a^ *R* − *sym* = (∑_*h*_∑_*i*_|*I*_*i*_(*h*) − < *I* (*h*) > |) / (∑_*h*_∑_*I*_ *I* (*h*)), where I_i_(h) is the i^th^ measurement of reflection h and < I(h) > is the weighted mean of all measurements of h. ^b^5 % of the reflections were used in the R-free calculations.

Like other bacterial β-clamps, the *Dr*β-clamp forms a head to tail dimer of two monomers, each monomer composed of three domains with identical α/β topology (Figure 1). The domains are slightly shifted in position when comparing the two dimers, so that in addition to NCS restrains between chains A and B we also used TLS refinement where each domain was defined as a separate group. The final RMSD between the chains is 0.9 Å and between the domains on average 1.8 Å. Compared to the *Ec*β-clamp the overall RMSD is 3.3 Å.

**Figure 2:**
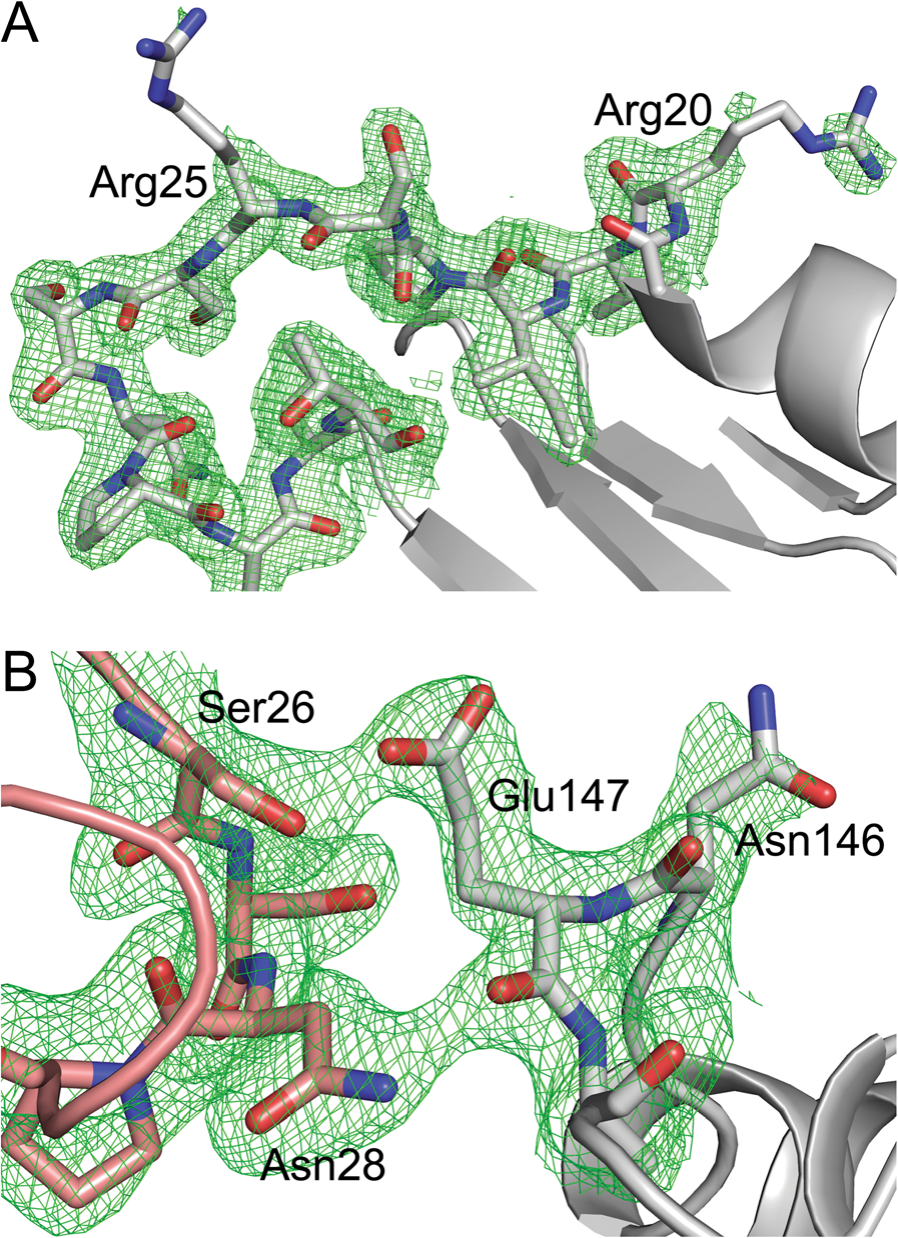
**Electron density (in green, contoured at** **σ = 1.0) around selected** ***Dr*** **β** **-clamp residues. (A)** Loop 23–32 (between α1 and β2) has clearly defined density except for the side chains of Arg20 and Arg25. **(B)** In loop 144–149 (in grey, between α3 and β11) Glu147 makes contact with Ser26 and Asn28 of a neighbouring clamp molecule.

The shape of the *Dr*β-clamp dimer is slightly more elliptic than that of the circular *Ec*β-clamp, with an internal diameter of 32 to 41 Å. A comparison of *Dr*β-clamp core structure and surface contour (Figure 1, left panel) suggests that the elliptic shape of *Dr*β-clamp cavity is enhanced by the conformation of certain long side chains and loops. The positively charged residues on the ring inner surface (Figure 1, right panel) appear flexible and side chains not involved in inter- or intramolecular interactions display poor electron density (Figure 2). In *Ec*β-clamp many of the basic side chains inside the clamp ring have been found to display flexibility and become ordered first upon contact with DNA [12].

### Surface potential

**Figure 3:**
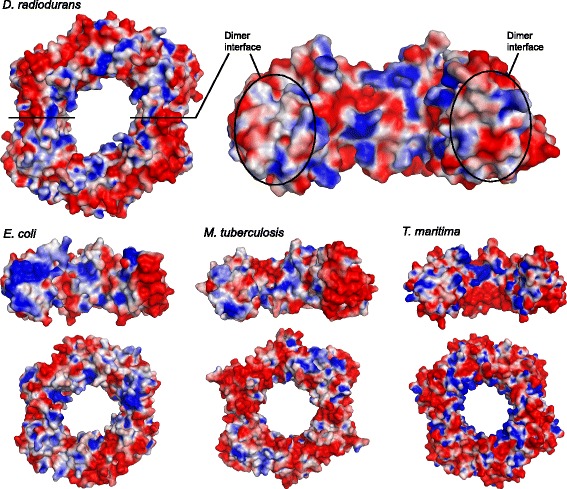
**Electrostatic surface potential of** **β-clamps from** ***D. radiodurans*** **(this work),** ***E. coli (*** **PDB code 1POL),** ***M. tuberculosis*** **(PDB code 3P16) and** ***T. maritima (*** **PDB code 1VPK).** The dimer molecules are depicted with the C-terminal protein-interacting side facing up (dimer), and the monomers showing the inside of the half-ring and the dimer interfaces. The surface is coloured according to the electrostatic potential at 298 K (−8 to 8 kT/e) with negative potential in red and positive potential in blue.

A comparison of the electrostatic surface potential of β-clamps from *D. radiodurans*, *E. coli*, *Mycobacterium tuberculosis* and *Thermotoga maritima* (Figure 3) reveals some interesting differences. All molecules have a more or less uniform negative charge on the outside of the ring, with this effect being strongest in *T. maritima* and weakest in *E. coli*. On the inside of the ring all molecules have positively charged residues for interacting with DNA. In *E. coli*, *M. tuberculosis* and *T. maritima* clamps the positive charge forms a relatively continuous band pattern across the surface whereas in *Dr*β-clamp the charge is more spread forming small positive patches separated by negatively charged areas. The surface charge distribution of *Dr*β-clamp may suggest that DNA binding is less tight or less specific compared to the other clamps. An even distribution of both positive and negative charge may facilitate efficient clamp sliding on DNA by hindering the formation of strong local interactions that might slow down the sliding movement.

The *Dr*β-clamp dimer interfaces have a spread, even charge distribution compared to the very strong positive (N-terminal domain) and negative charge (C-terminal domain) of the *Ec*β-clamp interfaces. The *M. tuberculosis* and *T. maritima* interfaces fall in between these two opposites, with *T. maritima* resembling more *Dr*β-clamp. In *T. maritima* β-clamp and *Dr*β-clamp electrostatic interactions may be less important in dimer formation and stability. Analysis of *Dr-* and *Ec*β-clamp interfaces with the Protein Interfaces, Surfaces and Assemblies service (PISA; [22]) shows that the *Ec*β-clamp interface is larger and has more hydrogen bonds and ionic interactions (24 and 7, respectively) compared to *Dr*β-clamp (15 and 2 interactions, respectively). The effect of dimer interface electrostatic interactions on clamp loading and dimer stability is currently not known.

### The hydrophobicity pocket

**Figure 4:**
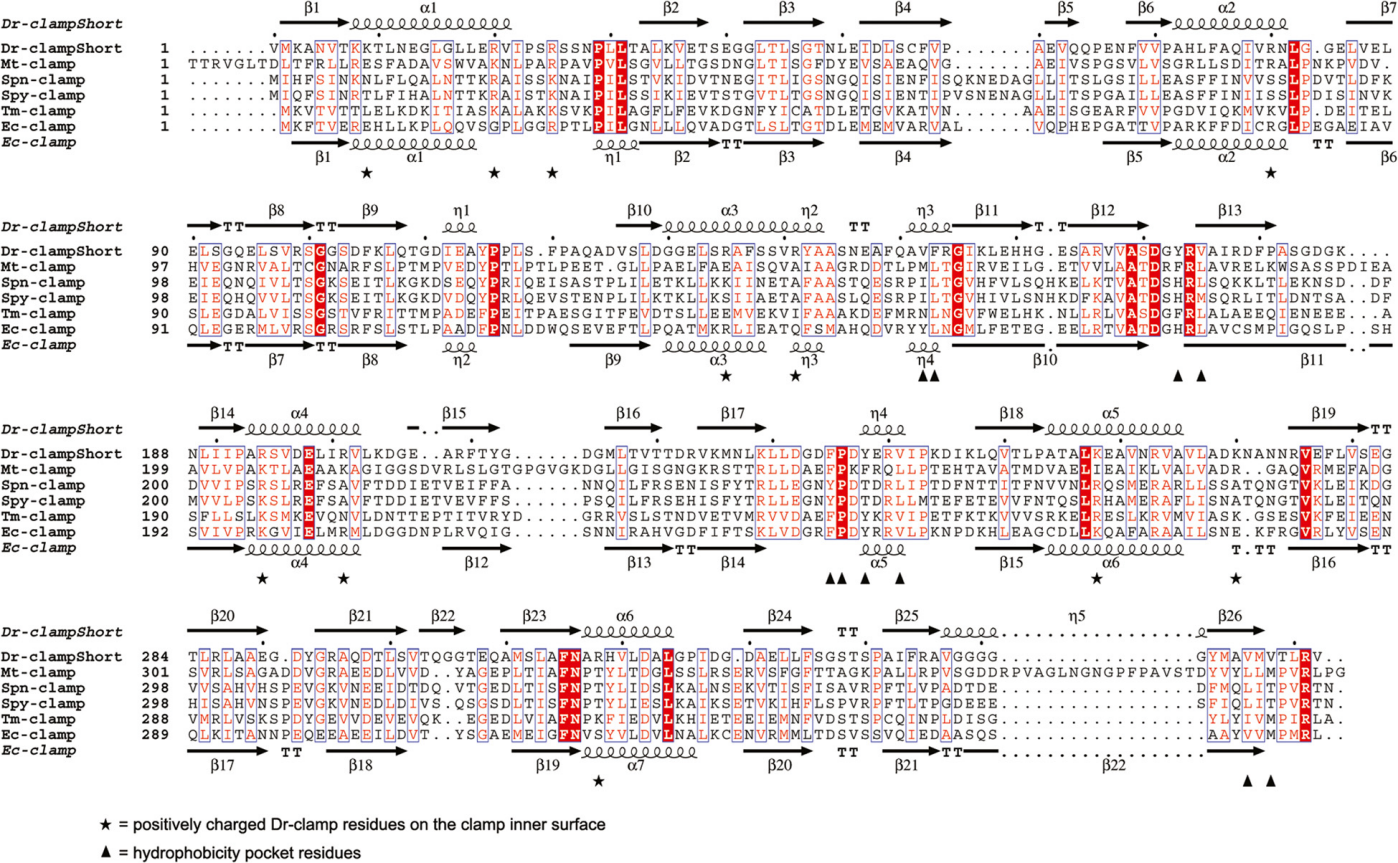
**Sequence alignment of the bacterial β-clamps with determined crystal structures.** *Dr*β-clamp short, *Mycobacterium tuberculosis* (*Mt*β-clamp; Gui et al., [7]), *Streptococcus pneumoniae* clamp (*Spn*β-clamp; unpublished, PDB code 2AWA), *Streptococcus pyogenes* clamp (*Spy*β-clamp; Argiriadi et al., [6]), *Thermotoga maritima* clamp (*Tm*β-clamp; unpublished, PDB code 1VPK), and *Ec*β-clamp (Kong et al., [5]). Stars denote the positively charged *Dr*β-clamp residues facing the inside of the ring, and triangles indicate hydrophobicity pocket residues. The figure was prepared with ESPript 3.0 [24].

A groove lined with hydrophobic residues called the hydrophobicity pocket or protein interaction pocket is located between domains B and C in *Ec*β-clamp. This pocket has been shown to serve as a ssDNA interaction site during clamp loading [12] in addition to being important for protein-protein interaction [23]. An analysis of the amino acid content (Figure 4) and structure (Figure 1, right panel) between domains B and C in *Dr*β-clamp suggests that this hydrophobicity pocket is present also in *Dr*β-clamp which thus may serve the same function as in *Ec*β-clamp.

### DNA interacting residues

The positions of positively charged residues on the β-clamp inner surface appear to be only moderately conserved (Figure 4), indicating that DNA backbone positioning is not critical. The DNA complex structure of *Ec*β-clamp identified two residues inside the β-clamp ring, Arg24 and Gln149, which are important for dsDNA interaction and necessary for clamp loading [12]. In *Dr*β-clamp these correspond to Arg25 and Glu147 (Figure 2). The consequence of having a negatively charged residue in *Dr*β-clamp in the same position as Glu147 in *Ec*β-clamp is not known, since no protein-DNA interaction data is available at the moment. However, this residue is completely conserved among the sequenced *Deinococcus* species, and is found also in *M. tuberculosis* and *T. maritima* β-clamps, and probably serves a similar function in these organisms as Glu147 in *Ec*β-clamp.

### Structural explanation for lack of DNA binding

An analysis of the *Ec*β-clamp-DNA complex suggests that the DNA in the structure is oriented in the opposite direction from what could be expected. This is caused by an interaction between the 5′ end of the DNA (4 T) and the hydrophobicity pocket of a neighbouring clamp molecule. Although this interaction is biologically relevant, it should normally occur on the same molecule which the DNA is inside. The strong interaction with the hydrophobicity pocket is caused by stacking interactions of the third thymidine and the first adenine base of the oligomer with two tyrosines (Tyr153 and Tyr154) in the pocket [12]. In *Dr*β-clamp these tyrosines are substituted by Ala151 and Val152, which may explain why the DNA is bound in a too disorganised fashion to be clearly visible in the electron density. The electron density for DNA is weak also in the eukaryotic complex structure [13], which may indicate that the true DNA binding mode is too unspecific and flexible to be well recorded by crystallographic methods. Co-crystallization with different length and type oligonucleotides should be tested to screen for more specific binding enabling DNA visualization.

## Conclusion

We have determined the crystal structure of *Dr*β-clamp to 2.0 Å resolution. The protein is a ring shaped dimer with a head-to-tail orientation and is similar to previously determined structures of bacterial β-clamps. Based on the observation of an even charge distribution inside the ring we hypothesise that the protein is optimised for efficient function during high turnover of DNA metabolic processes.

## Methods

### Cloning, expression and purification

The gene encoding *Dr*β-clamp (*DR_0001*) was cloned from genomic DNA of *D. radiodurans* strain R1 into expression vector pDEST14 (Invitrogen). All primers used in cloning are listed in Table 2. We used a two-step Gateway method with gene specific primers Fw1 and Rev1 which introduced a TEV-cleavable His_7_-tag to the C-terminus of the protein, and extension primers *att*B1 and *att*B2 that contained the rest of the *att*-sites. During sequence verification it was discovered that the cloned gene contained a deletion of cytosine 1039 compared to the published genomic sequence of *D. radiodurans* strain R1 [25]. Bioinformatic analysis of the gene and protein sequence of *Dr*β-clamp showed that the absence of cytosine 1039 leads to a frameshift and an earlier stop codon producing a shorter version of the gene (1089 nt compared to the 1182 nt GeneBank version) which encodes a 362 aa protein more similar both in length and in sequence to other known β-clamps (Figure 4) than the longer version. We cloned both the short and the long version of the gene to see if they could both be expressed. We recreated the long version by reintroducing the missing cytosine 1039 to the already cloned, deletion containing gene by site-directed mutagenesis using the QuikChange kit from Stratagene (primers Ins-fw and Ins-rev, Table 2). The short version was cloned from genomic DNA using the same procedure as for the long version, except that the primers were Fw1 and Rev2.

**Table 2:** **Cloning primers used in this work**

**Name**	**Sequence**
Fw1	5′-AAAAAGCAGGCTTCGAAGGAGATAGAACCATG**GTGATGAAAGCCAAT GTCACC**
Rev1	5′-AGAAAGCTGGGTCTTAGTGATGGTGATGGTGATGTCCCTGGAAATACA GGTTTTC**CGCGAACTCTGGCCTCGGTTC**
Rev2	5′-AGAAAGCTGGGTCTTAGTGATGGTGATGGTGATGTCCCTGGAAATACA GGTTTTC**AACGCGCAGCGTGACCATGACC**
attB1 adapter	5′-GGGGACAAGTTTGTACAAAAAAGCAGGCT
attB2 adapter	5′-GGGGACCACTTTGTACAAGAAAGCTGGGT
Ins-fw	5′-CATTTTCCGCGCC**C**GTAGGTGGGGGA
Ins-rev	5′-TCCCCCACCTAC**G**GGCGCGGAAAATG

Gene specific parts are in bold, except in mutagenesis primers where bold indicates the inserted base.

Only the short version of *Dr*β-clamp could be successfully expressed and was verified by MS peptide fingerprinting. The *Dr*β-clamp was expressed in *E. coli* BL21(DE3)Star pLysS pRARE (Invitrogen) with 0.5 mM IPTG induction overnight at 293 K. The cells were suspended in 50 mM Tris, 150 mM NaCl, pH 7.5, and disrupted by sonication followed by centrifugation at 20 000xg for 25 min, 277 K. The protein was purified with affinity and ion exchange chromatography (HisTrap HP and HiTrap Q columns from GE Healthcare), and the His_7_-tag was cleaved off by incubating the protein with 1/50 w/w TEV protease with 1 mM DTT and 0.5 mM EDTA added. Unprocessed protein was removed by using a HisTrap HP column in flow through mode, and an Amicon Ultra Centrifugal Filter (10 000 MWCO, Millipore) was used for concentration of the protein and buffer exchange to the original conditions. For size and tertiary structure analysis *Dr*β-clamp was run on Superdex 75 10/30 (GE Healthcare) where it behaved as a dimer of approximately 80 kDa.

### Oligonucleotide annealing and purification

We obtained from Sigma-Genosys two unlabelled DNA oligos (5′-TTTT ATACGATGGG, 5′-TTTTTT ATACGATGGG) and one Cy5-labeled oligo (5′-Cy5-CCCATCGTAT) in order to create two different Cy-5 labelled double-stranded oligos with 10 nt double-stranded region and either 4 or 6 nt long single-stranded thymidine overhang (4 T and 6 T). This method was previously used successfully by both [12] and [13]. The oligos were dissolved in 50 mM HEPES, 50 mM NaCl and 0.5 mM EDTA, pH 8.0, the labelled oligos mixed with a slight excess of unlabelled oligos (1:1.1), and annealed by placing in a 343 K heat-block and allowed to cool to room temperature overnight. The annealed oligos were purified with a GE Healthcare Mono Q HR 5/5 column, concentrated with an Amicon 4 ml 3000 MWCO concentrator (Millipore) and dialysed overnight at 277 K in Pierce Slide-A-Lyzer MINI dialysis tubes (MWCO 2 000) against 50 mM HEPES pH 8.0. Oligomer concentration was measured with a NanoDrop 2000c spectrophotometer (NanoDrop Technologies) at 650 nm using the extinction coefficient for Cy5 (2.5×10^5^ M^−1^ cm^−1^).

### Crystallisation and data collection

**Figure 5:**
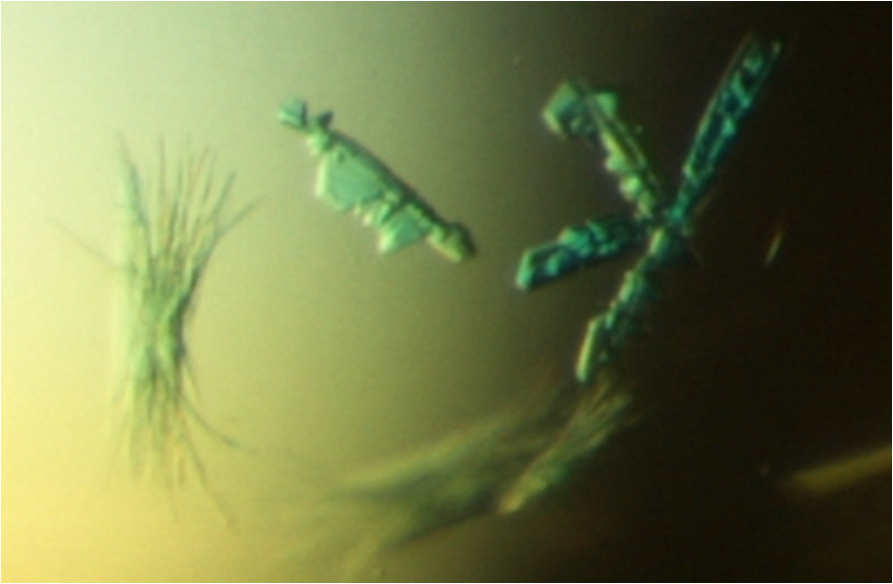
**An image of the blue** ***Drβ*** **-clamp crystals obtained with 4 T-DNA oligo.** Pure *Dr*β-clamp was mixed with purified Cy5-labeled 4 T oligos in a 1:1.1 protein:DNA ratio. Crystallization in 21% PEG 5000 MME, 0.12 M Tris, 3.6% hexanediol, pH 8.0, using the 4 T oligo containing protein solution yielded blue needles and clusters of small blue crystals.

For crystallisation, pure *Dr*β-clamp was mixed with purified Cy5-labeled 4 T/6 T oligos to give a solution of 5 mg/ml protein with 1:1.1 protein:DNA ratio. Crystallisation was done in sitting-drop format using a Crystal Phoenix liquid dispenser (Art Robbins Instruments) with MRC-2 plates (Molecular Dimensions). Home-made stochastic screens were used for initial screening. Clusters of small blue crystals (Figure 5) were obtained in conditions with 21% PEG 5000 MME, 0.12 M Tris, 3.6% hexanediol, pH 8.0, using the 4 T oligo containing protein solution. A strong blue colour indicated that the crystals contained DNA. Single crystals were separated from the clusters, transferred into a cryo solution containing 23% PEG 5000 MME, 0.15 M Tris, 4% hexanediol, and 19% glycerol, pH 8.0, and immediately flash-frozen in liquid nitrogen. Diffraction data were collected on a single crystal at the European Synchrotron Radiation Facility (ESRF), Grenoble, France, at beamline ID-29 equipped with a Pilatus 6 M detector [26] at 100 K to 2.0 Å.

### Structure determination and refinement

The data were indexed, integrated, scaled and converted to structure factors using the XDS program package [27]. The space group P3_2_21 was chosen after analysis with the program Pointless [28]. The unit cell dimensions are a = b = 84.66 Å and c = 199.08 Å. We found two monomers (one β-clamp dimer) in the asymmetric unit, giving a solvent content of 53.8% and a Matthews coefficient of 2.66 Å^2^/Da. The structure was solved by molecular replacement using MOLREP [29] in CCP4 [30] with the *Ec*β-clamp (PDB code 2POL, 29% identity) as a search model. Because of the relatively low identity between the model and *Dr*β-clamp, ARP/wARP tracing [31] was used to find the correct backbone position. The structure was refined in REFMAC5 [32] using TLS refinement where each of the three domains of a monomer was defined as a group, and automatically determined NCS restraints (chain A to B). Inspection and manual building of the model between refinement runs was done using Coot [33], and water molecules were added using Coot findwaters. During the refinement it became obvious that although the crystals contained DNA, the observed electron density for it (in the central cavity of the β-clamp ring) was too weak to support the placement of any nucleotides in the model.

### Structure analysis

The structural model quality, geometry and fit to electron density were evaluated using Coot tools, and finally validated with the program MolProbity [34]. Structural images were drawn with PyMol (http://www.pymol.org/) and the APBS plugin [35] was used for calculation of the electrostatic surface potentials.

## Abbreviations

β-clamp: DNA polymerase III β subunit
*Dr*β-clamp: *D. radiodurans* β-clamp
PCNA: Proliferating cell nuclear antigen
*Ec*β-clamp: *E.coli* β-clamp
ESRF: European Synchrotron Radiation Facility
RMSD: Root mean square deviation
